# Ischemic polypectomy for small-bowel polyps in pediatric Peutz-Jeghers syndrome

**DOI:** 10.1016/j.vgie.2025.09.006

**Published:** 2025-10-08

**Authors:** Jared M. Grabau, Umer Bhatti, Brett J. Hoskins

**Affiliations:** 1 Department of Medicine, Marian University Tom and Julie Wood College of Osteopathic Medicine, Indianapolis, Indiana, USA; 2 Division of Gastroenterology and Hepatology, Department of Medicine, Indiana University School of Medicine, Indianapolis, Indiana, USA; 3 Division of Pediatric Gastroenterology, Hepatology and Nutrition, Department of Pediatrics, Riley Hospital for Children, Indiana University School of Medicine, Indianapolis, Indiana, USA

## Abstract

**Background and Aims:**

Peutz-Jeghers syndrome (PJS) is characterized by the development of hamartomatous polyps in the gastrointestinal tract that can cause chronic blood loss. Ischemic polypectomy has emerged as a low-risk technique in adult patients with PJS but remains largely undocumented in pediatric populations. This video presents the first published case demonstrating ischemic polypectomy in a pediatric patient, to our knowledge.

**Methods:**

A 12-year-old girl with PJS and chronic anemia underwent balloon-assisted enteroscopy. Seven pedunculated small-bowel polyps were identified and treated using ischemic polypectomy with a detachable snare.

**Results:**

Three representative polypectomies are shown. The snare was tightened at the polyp stalk until ischemic changes occurred, then cinched and deployed. One instance required a second snare because of equipment malfunction. All polyps were successfully treated without electrocautery, bleeding, or perforation. Follow-up demonstrated improvement in longstanding anemia, with a rise in hemoglobin from 9.1 to 12.4 g/dL at 6 weeks, supporting therapeutic benefit. Repeat capsule endoscopy was planned.

**Conclusions:**

Ischemic polypectomy using balloon-assisted enteroscopy is feasible, effective, and safe in pediatric PJS. By avoiding thermal energy, it may reduce the risk of transmural injury and represents a valuable option for children with numerous pedunculated small-bowel polyps.

## Introduction

Peutz-Jeghers syndrome (PJS) is a rare genetic disorder characterized by mucocutaneous pigmentation and hamartomatous polyps throughout the gastrointestinal tract. These polyps may lead to significant morbidity, including bleeding, anemia, intussusception, and obstruction.^1^ Endoscopic polypectomy is essential for management; however, standard techniques carry a higher risk of perforation and bleeding in the thin-walled, highly vascular small bowel, where electrocautery can more easily cause deep thermal injury.^1^^,^^2^ This risk is further amplified in pediatric patients, whose thinner intestinal wall, narrower lumen, and smaller, more-mobile abdominal cavity create additional technical challenges, particularly when adult-sized enteroscopes and accessories are used.^3^, ^4^, ^5^ Ischemic polypectomy, which involves mechanical strangulation of the stalk to induce autoamputation, has been successfully used in adults with PJS.^6^^,^^7^ In pediatrics, the approach is rarely described,^8^ and no visual documentation currently exists. This case represents the first published video demonstration of ischemic polypectomy in a pediatric patient, to our knowledge.

## Case presentation

A 12-year-old girl with genetically confirmed PJS and chronic iron deficiency anemia underwent therapeutic balloon-assisted enteroscopy following capsule endoscopy, which identified multiple pedunculated small-bowel polyps. Antegrade single-balloon enteroscopy with the patient under general anesthesia revealed 7 pedunculated polyps (10-20 mm) in the duodenum and jejunum, all treated using ischemic polypectomy (Video 1, available online at www.videogie.org).

### Technique

Ischemic polypectomy was performed using a PolyLoop detachable ligating device (HX-400U-30; Olympus America Inc, Center Valley, Pa, USA) during single-balloon enteroscopy (SIF-Q180; Olympus Medical Systems, Tokyo, Japan). After isolation of each polyp, the detachable snare was carefully positioned at the base of the stalk and slowly tightened until a dusky purple color change indicated vascular compromise. The snare was then cinched and deployed, allowing the polyp to remain in situ for autoamputation. Although not used in this case, hemostatic clips applied in a cross configuration at the stalk base have also been described to induce ischemia.^6^ Potential risks of this approach include delayed bleeding, device failure, and incomplete ischemia, although these may be offset by the reduced risk of electrocautery-related perforation. A key limitation is the inability to retrieve polyps for histology; however, in confirmed PJS, tissue sampling is less critical for diagnosis, and targeted biopsy specimens can be obtained from suspicious areas if needed.^2^

**Figure 1:**
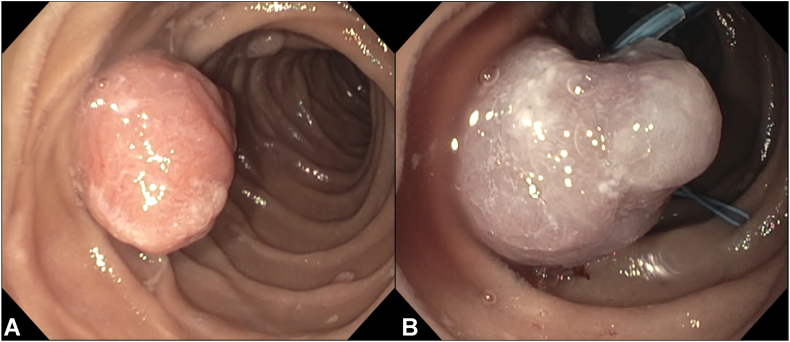
Endoscopic images from ischemic polypectomy in a 12-year-old girl with Peutz-Jeghers syndrome: (**A**) 20-mm pedunculated jejunal polyp prior to intervention; (**B**) same polyp after deployment of a detachable snare on the stalk, demonstrating dusky discoloration consistent with vascular strangulation and early ischemia.

In 1 example shown in the video (Video 1), the initial detachable snare failed to deploy properly despite initial color change, resulting in reperfusion of the polyp. A second detachable snare was placed on the same stalk, leading to successful ischemia and secure deployment (Fig. 1). This highlights the importance of visual confirmation of sustained ischemia and readiness to reattempt in case of device malfunction.

### Outcome and follow-up

The procedure was well tolerated without immediate or delayed adverse events. At follow-up, the patient's chronic anemia had improved (hemoglobin improved from 9.1 g/dL to 12.4 g/dL). Autoamputation of treated polyps was expected within 2 to 3 weeks, with planned follow-up capsule endoscopy imaging in 2 to 3 months.

## Discussion

This case illustrates the feasibility of ischemic polypectomy in pediatric patients with PJS using balloon-assisted enteroscopy. This technique offers several advantages, including avoidance of thermal injury, procedural simplicity, and improved safety in the small bowel. Pediatric patients present unique technical challenges during balloon-assisted enteroscopy, including a smaller, more-mobile abdominal cavity, thinner intestinal wall, and narrower lumen.^3^, ^4^, ^5^ These factors necessitate careful technique and preprocedure planning to safely identify and manage clinically significant small-bowel polyps.

The example of incomplete initial deployment reinforces the need for both visual and technical confirmation of ischemia. In that instance, a malfunctioning snare led to revascularization of the polyp, which was successfully addressed with a second deployment on the same stalk.

Although ischemic polypectomy has been previously described in adults, this is the first published video demonstrating its use in a pediatric patient, to our knowledge. This case expands the therapeutic options available to pediatric endoscopists managing high-risk small-bowel polyps in children with PJS.

## Conclusion

Ischemic polypectomy is a feasible and safe technique for managing pedunculated small-bowel polyps in pediatric PJS. When performed with careful technique and visual confirmation of sustained ischemia, it may offer a lower-risk alternative to traditional thermal and cold resection methods. This case provides video-based technical insights to support broader adoption of the technique in appropriate pediatric patients.

## Patient Consent

Written informed consent was obtained from the patient's legal guardian for publication of this case and accompanying images and video.

## Disclosure/Funding

The following author disclosed financial relationships: B. J. Hoskins: Consultant: Mirum Pharmaceuticals Inc and 3-D Matrix Inc. All other authors disclosed no financial relationships.

## Supplementary data

Video 1 This video demonstrates ischemic polypectomy of 3 pedunculated small-bowel polyps in a 12-year-old girl with Peutz-Jeghers syndrome using single-balloon enteroscopy. A detachable snare was placed tightly at the polyp stalk until dusky discoloration was seen, then cinched and deployed without need for electrocautery. One instance illustrates repeat snare placement due to incomplete initial deployment with reperfusion of the polyp. Proper technique resulted in successful ischemia and no adverse events.
